# Contrasting Mode of Evolution at a Coat Color Locus in Wild and Domestic Pigs

**DOI:** 10.1371/journal.pgen.1000341

**Published:** 2009-01-16

**Authors:** Meiying Fang, Greger Larson, Helena Soares Ribeiro, Ning Li, Leif Andersson

**Affiliations:** 1 Department of Animal Breeding and Genetics, Swedish University of Agricultural Sciences, Uppsala, Sweden; 2 College of Animal Science and Technology, China Agricultural University, Beijing, China; 3 Department of Medical Biochemistry and Microbiology, Uppsala University, Uppsala, Sweden; 4 State Key Laboratory for Agrobiotechnology, China Agricultural University, Beijing, China; Stanford University School of Medicine, United States of America

## Abstract

Despite having only begun ∼10,000 years ago, the process of domestication has resulted in a degree of phenotypic variation within individual species normally associated with much deeper evolutionary time scales. Though many variable traits found in domestic animals are the result of relatively recent human-mediated selection, uncertainty remains as to whether the modern ubiquity of long-standing variable traits such as coat color results from selection or drift, and whether the underlying alleles were present in the wild ancestor or appeared after domestication began. Here, through an investigation of sequence diversity at the porcine *melanocortin receptor 1* (*MC1R*) locus, we provide evidence that wild and domestic pig (*Sus scrofa*) haplotypes from China and Europe are the result of strikingly different selection pressures, and that coat color variation is the result of intentional selection for alleles that appeared after the advent of domestication. Asian and European wild boar (evolutionarily distinct subspecies) differed only by synonymous substitutions, demonstrating that camouflage coat color is maintained by purifying selection. In domestic pigs, however, each of nine unique mutations altered the amino acid sequence thus generating coat color diversity. Most domestic *MC1R* alleles differed by more than one mutation from the wild-type, implying a long history of strong positive selection for coat color variants, during which time humans have cherry-picked rare mutations that would be quickly eliminated in wild contexts. This pattern demonstrates that coat color phenotypes result from direct human selection and not via a simple relaxation of natural selective pressures.

## Author Summary

This study addresses why coat colors of domestic animals are so variable, while those of their wild ancestors are so uniform. Specifically, we asked whether this was the result of (i) relaxed purifying selection, (ii) that some mutations affect both coat color and another trait under strong selection (for instance behavior), or (iii) direct human selection for altered coat color phenotypes. We investigated genetic variation in the *melanocortin receptor 1* (*MC1R*) gene among wild and domestic pigs from both Europe and Asia. Though we found a similar number of mutations in wild and domestic pigs, the nature of the mutations was strikingly different. All mutations found among wild boars were silent, i.e., they did not change the protein sequence. This implies strong purifying selection in the wild that maintains camouflage coat color. In contrast, nine out of ten mutations found in domestic pigs altered the protein sequence, thereby drastically transforming the resulting coat color. These results demonstrate that early farmers intentionally selected pigs with novel coat coloring. Their motivations could have been as simple as a preference for the exotic or selection for reduced camouflage to facilitate animal husbandry and/or to make the domesticated forms distinct from their wild ancestor.

## Introduction

The sizes, shapes, and colors among domestic animals vary significantly more than that of their wild counterparts, often reflecting variation normally associated with genus or family level divergence [1]. Domestication therefore provides an ideal model to test numerous evolutionary questions including the relationship between molecular and morphological change, how the intensification of the relationship between humans and wild plants and animals have altered both players' genetic and phenotypic constitutions, and whether changes associated with domestication resulted primarily from a release of natural selection pressure, selection on standing genetic variation present in the wild ancestor, or positive selection on novel mutations that have occurred subsequent to domestication.

Coat color variation in domestic animals is of considerable interest in this respect considering that it can be traced back to at least 5,000 years before present when it was documented by administrative officers who recorded the coat color of livestock during the UR III dynasty in Mesopotamia [2]. Modern domestic animal species display a bewildering diversity in coat color, and the *melanocortin receptor 1* (*MC1R*) locus is most consistently polymorphic, having been previously documented and associated with coat color variation in horses, cattle, foxes, pigs, sheep, dogs, and chickens [3]–[10].

MC1R is a G protein-coupled receptor that is primarily expressed in melanocytes and plays a key role in melanogenesis by determining the switch between production of red/yellow pheomelanin and dark eumelanin [11]. The binding of melanocyte stimulating hormone (MSH) to MC1R induces synthesis of eumelanin, whereas in the absence of MC1R signaling, melanocytes produce only pheomelanin. Loss-of-function mutations are therefore associated with recessive red coat color, whereas dominant black coloring is linked with mutations causing constitutive activation of MC1R signaling.

We have previously described the molecular basis for an allelic series at the classical *Extension* locus (equivalent to *MC1R*) in pigs [6],[7],[12]. The wild-type (*E^+^*) allele allows full expression of both pheomelanin and eumelanin. The dominant black color results from two different mutations, each of which evolved independently in Asia and Europe. The *E^D1^* allele is Asian in origin and is associated with an L102P missense mutation, and *E^D2^* is European and associated with a D124N substitution. The recessive red allele (*e*) possesses two missense mutations A164V and A243T, though it is not clear if one or both of these are responsible for the phenotype.

The most interesting allele is *E^P^* which causes black spotting on a red or white background. This allele evolved from *E^D2^* and possesses, in addition to the D124N substitution for dominant black coloring, a two base pair insertion at codon 22. Two C nucleotides have been inserted in a stretch of six Cs, thus extending a short mononucleotide repeat. The resulting frameshift is expected to cause a uniform red pigmentation due to the complete loss of MC1R signaling, but because the mononucleotide repeat is somatically unstable, MC1R function in some melanocyte lineages is occasionally restored, resulting in the appearance of black spots [7]. All of these alleles were described from a limited subset of wild and domestic pigs.

In order to more fully understand the range of *MC1R* variations within *Sus scrofa*, we determined the entire *MC1R* coding sequence (963 bp) within 68 domestic pigs (representing 51 Asian and European breeds) and a total of 15 Chinese and European wild boar, the results of which are described in Tables S1, S2, S3, S4. In addition, one previously published Japanese wild boar sequence [13] was incorporated into the analysis.

## Results

The sampling strategy outlined above resulted in a near doubling of the number of described *MC1R* alleles from seven to 13, all of which belonged to the five previously described allelic groups. A large proportion of the domestic pigs (60 out of 68) were homozygous at *MC1R* despite the fact that eight different alleles were identified within domestic pigs (Table S3). This high degree of homozygosity is not surprising given that coat colors have been used as a specific breed characteristic over the past 200 years, and within most populations there has been strong selection for uniformity in coat color as this trait often defines the breed.

This screen revealed three new missense mutations in pig MC1R, Val122Ile in the Asian *0202 allele, Ala21Thr in the European *0502 allele and finally Arg166Trp in the European *0503 (Table S2). Since these variants were detected in a few pigs that may carry other coat color mutations we cannot judge if they have an impact on the coat color phenotype and this needs to be further investigated. Until such data become available we assume that they are associated with dominant black color (*0202) and black spotting (*0502 and *0503).

All domestic breeds in Europe and China carried mutant *MC1R* alleles except the Hungarian Mangalica. Though this breed is homozygous for the European wild type *MC1R* allele, it still possesses a variable coat color phenotype, a fact at least partially explained by allelic segregation at the agouti (*ASIP*) locus [14]. The *E^P^* (black spotting) *MC1R* allele dominates among European domestic pigs, particularly amongst commercial populations (Table S4). The allele for dominant black color (*E^D2^*) is more common in local European breeds whereas the allele (*e*) for recessive red color was only found in the Duroc and Leicoma breeds. One Creole pig carried the Asian *E^D1^* allele for dominant black color, reflecting the introgression of Asian pigs into European stocks that took place during the 18^th^ and 19^th^ century [13]. All tested Chinese pigs carried the *E^D1^* allele for dominant black color. Lastly, three Chinese domestic pigs were heterozygous for the European *e* or *E^P^* alleles, an observation that is most certainly caused by recent introgression of European germplasm into local Chinese breeds.

The European, Chinese, and Japanese wild boars all carried wild-type alleles that differ only by synonymous substitutions (Tables S2, S3). All 12 European wild boar carried identical *MC1R* sequences which were also identical to a previously described European wild boar sequence [13] and to the one present in Mangalica domestic pigs. The complete absence of synonymous substitutions between European wild boar and European domestic pig *MC1R* sequences supports the notion that the European wild boar experienced a population bottleneck prior to domestication [15],[16]. The higher *MC1R* diversity within Chinese wild boar relative to European wild boar is also consistent with recent microsatellite data indicating that Asian domestic pigs were derived from a more diverse wild boar population [17]. One Chinese wild boar was heterozygous for the *E^P^* allele but this must be the result of gene flow from domestic pigs since the allele is of European origin and differs from native Chinese wild boar alleles by at least two synonymous and two non-synonymous substitutions (Table S2).

**Figure 1:**
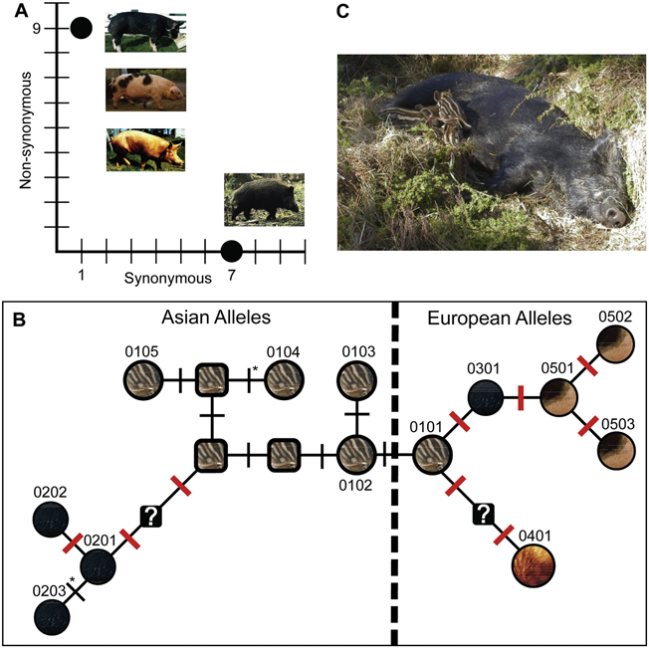
Amino acid sequence conservation within wild boar is sharply contrasted by the sequence diversity amongst *MC1R* alleles found within domestic pigs. (A) The observed number of synonymous and non-synonymous substitutions amongst Chinese and European domestic pigs and amongst Chinese and European wild boars; included amongst the “non-synonymous” substitutions is the two base pair insertion associated with the *E^P^* allele. The diversity of coat color among domestic pigs is exemplified by the images on the left and contrasted with the camouflaged coat color in the wild boar on the lower right. (B) A Median-joining network of *MC1R* alleles in Asian and European pigs. All known alleles are represented by circles while squares represent predicted intermediate forms that have not yet been found. Thin black lines placed perpendicular to the lines connecting the individual haplotypes represent single, synonymous changes, and thicker red lines represent non-synonymous changes. The asterisk placed near the two black lines leading to alleles *0104 and *0203 indicates the only instance of an identical mutation at different locations on the tree. Colors inside the circles and squares depict the observed and predicted coat colors associated with a given allele; question marks inside two of the squares indicate that the associated coat color of these intermediate alleles cannot be predicted because they connect alleles that differ by two non-synonymous substitutions. The first two digits in the nomenclature for porcine *MC1R* alleles alongside the circles indicate the associated coat color phenotype: 01 = wild type, 02 = dominant black, Asian form, 03 = dominant black, European form, 04 = recessive red, and 05 = black spotting. The two last digits are used to distinguish alleles that are associated with the same phenotype. (C) Wild boar sow with piglets in the nest. Note the remarkable camouflage coat color pattern in the piglets. (Photo: Anneli Andersson, Linköping University, Sweden.)

The amino acid sequence conservation within wild boar is sharply contrasted by the sequence diversity amongst *MC1R* alleles found within domestic pigs. This is illustrated in Figures 1A and 1B which depict the completely synonymous nature of all seven nucleotide substitutions found among European and Asian wild boar. The figure also shows that all mutations except one detected amongst European and Chinese domestic pigs altered the amino acid sequence, eight are non-synonymous substitutions and one is the frameshift at codon 22 that is widespread among European domestic pigs. The same synonymous substitution was found in both the Chinese domestic allele *0203 and in the Asian wild boar allele *0104 (Figure 1B).

The existence of radically different selective pressures acting on *MC1R* in wild and domestic pigs was demonstrated by an analysis of the relative frequencies of non-synonymous (dN) and synonymous substitutions (dS) (Table 1); a dN/dS ratio higher than 1.0 indicates positive selection while a dN/dS ratio significantly lower than 1.0 provides evidence for purifying selection. The most informative contrasts were those between Asian and Europan wild boars and between Asian and European domestic pigs. The former comparison provided evidence for purifying selection in wild boars since no non-synonymous substitution was observed and the frequency of synonymous substitutions was significantly different from 0 (Z = 2.55, P<0.01). In contrast, the dN/dS ratio was as high as 23.5 for the comparison between Asian and European domestic pigs, ignoring the three synonymous substitutions that distinguish Asian from European alleles but which must have occurred in wild boars prior to domestication (Figure 1B). The excess of non-synonymous substitutions between European and Asian domestic pigs was statistically significant (Z  = 2.05; P<0.01) and provided strong support for positive selection. This analysis in fact underestimates the evidence for diversifying selection since the dN/dS analysis did not take into account the frameshift mutation that is widespread among European domestic pigs.

**Table 1:** Analysis of the frequency of non-synonymous (dN) and synonymous (dS) substitutions among wild and domestic pigs from Europe and Asia.

Comparison	n	%dN±s.e.	%dS±s.e.	dN/dS
Within populations				
European wild boar (EWB)	24	0	0	0
European domestic (ED)	89	0.13±0.06	0	∞
Asian wild boar (AWB)	5	0	1.33±0.51	0
Asian domestic (AD)	42	0.01±0.01	0.04±0.03	0.25
Between populations				
EWB vs ED		0.18±0.12	0	∞
AWB vs. AD		0.29±0.20	0.95±0.37	0.31
EWB vs. AWB		0	1.48±0.58	0
ED vs. AD^1^		0.47±0.23	0.02±0.02	23.5

n = number of sequenced chromosomes as indicated in Table S3; Asian alleles found in European pigs and European alleles found in Asian pigs were excluded from the analysis since they most certainly reflect recent introgressions.

1 The three synonymous substitutions present in both Asian wild boars and Asian domestic pigs (Table S2) were excluded from the analysis in order to restrict the analysis to those substitutions that occurred subsequent to domestication.

Furthermore, the large number of non-synonymous substitutions cannot be explained by relaxed selection subsequent to pig domestication. The reason is that the time since domestication (about 10,000 years) is not sufficiently long to accumulate as many mutations that have gone to fixation in domestic populations or the existence of several alleles that differ by multiple steps from the ancestral form. We have previously estimated the time since divergence of European and Asian wild boar populations to about 900,000 years before present based on a 1.2% sequence difference across the entire mitochondrial genome [18]. This is very similar to the recently estimated sequence divergence of about 1.3% for the entire mitochondrial genome between modern humans and Neandertals and the time since divergence of these two mtDNA lineages were estimated at 660,000±140,000 years [19]. Thus, the seven synonymous substitutions observed among Asian and European wild boars have accumulated during hundreds of thousands of years whereas the nine mutations changing the MC1R protein sequence among domestic pigs have accumulated within the last 10,000 years. Thus, we refute relaxed selection as a possible explanation for the fast evolution of *MC1R* diversity in domestic pigs and conclude that the only reasonable explanation for this plethora of diversity is that humans have cherry-picked novel mutations with favorable phenotypic effects during the course of domestication.

A reconstructed evolutionary history of porcine *MC1R* sequences (Figure 1B) demonstrates not only the pattern of synonymous substitutions (Table S2) that differentiate Asian and European wild boar sequences, but also the fact that domestic pig *MC1R* alleles are directly derived from wild boar from the same region, a conclusion supported by previous publications that investigated other loci [13],[18],[20].

## Discussion

The *MC1R* diversity within Europe and China presented here reveals divergent selection pressures in wild and domestic pigs. A previous study of mtDNA genome sequences derived from European and Asian wild boars indicated that these subspecies diverged well before the advent of domestication [18]. In this study, we identified a total of seven synonymous nucleotide substitutions within *MC1R* sequences from Asian and European wild boar. The temporally and geographically conserved nature of *Sus MC1R* amino acid sequence demonstrates that purifying selection is the dominant mode of evolution at this locus in the wild boar because normal *MC1R* function, which generates a mixture of dark eumelanin and red/yellow pheomelanin, is necessary for a camouflaged coat.

*MC1R* underlies coat color diversity in many natural and domestic populations. This study demonstrates that this diversity cannot be explained by an exceptionally high substitution rate at this locus. Instead, a more likely explanation of this pattern is that though *MC1R* is a master regulator for melanogenesis, its primary role is restricted to melanocytes. Thus, *MC1R* mutations can have major effects on coat color phenotypes without causing severe pleiotropic effects on other tissues, a phenomenon that often results from mutations at other coat color loci [21].

Because domestication necessarily involves the separation of animals from their natural environment, the alterations in coat color during animal domestication could have been the result of a relaxation of the selection pressure against non-camouflaged coat. The evidence presented here, however, demonstrates that relaxed selection alone cannot explain the observed *MC1R* diversity in domestic pigs. The complete lack of non-synonymous substitutions within European and Chinese wild boar (who diverged in the Pleistocene) is contrasted sharply by the nine separate amino acid-altering mutations present within domestic pigs. This pattern implies that naturally occurring mutations that altered camouflaged coat colors were quickly eliminated in the wild, but within a domesticated context, these mutations were prized and positively selected. Having been allowed to proliferate, the new mutated alleles served as templates on which additional mutations occurred (Figure 1B). This conclusion is underscored by the fact that seven of the eight *MC1R* alleles found in domestic pigs differed by two or more non-synonymous substitutions from the wild-type. In fact, three of the alleles (*0202, *0502 and *0503) differed by as many as three non-synonymous substitutions from the wild-type.

We propose that an important component of the selection at coat color loci in domestic animals has been direct selection for non-camouflaged patterns, since the mutated coat colors may have facilitated animal husbandry. The traditional practice of keeping domestic pigs involved allowing them to forage beyond the boundaries of the farm. Possessing a coat color significantly different from the wild type would have made it easier to recognize and keep track of domestic stocks. Furthermore, variant coat color phenotypes may also have been used as markers associated with improved domesticated forms.

Finally, the ubiquity of the black spotting caused by the *E^P^* allele, may be a result of the universal human penchant to select for and propagate attractive patterns present within the floral and fauna with which humans have a close relationship. Three recent illustrative examples of human preference for novelty include selection for white color in horses [22], the human-mediated spread of white Polynesian tree snails between Polynesian islands [23], and the global proliferation of white grape cultivars, which, like some domestic coat colors, results from the inactivation of a gene necessary for wild type (red) grape coloring [24].

Black coloring is common in both European and Asian domestic pigs but because different allelic substitutions underlie this indistinguishable phenotype, it is clear that there have been independent selective sweeps for separate alleles causing dominant black color in both China and Europe. In China, selection for the L102P substitution has been strong enough to penetrate all 23 breeds representing the six traditional types of Chinese pigs. Similarly, 90% of *MC1R* sequences found in European domestic pigs carried the D124N missense mutation causing dominant black color.

Despite dramatic phenotypic differences between wild and domestic animals, no fixed mutation has yet been identified that distinguishes domesticated and wild forms of a given species. The *MC1R* locus in pigs approaches such a diagnostic status since only one breed (the Mangalica) out of 51 included in this study carried the wild-type allele. Interestingly, to the best of our knowledge, Mangalica pigs are also the only pigs included in this study that give birth to striped piglets that resemble striped wild boar piglets (Figure 1C). This indicates that normal *MC1R* signaling is required for the development of stripes in piglets and it may therefore also be important for the development of stripes in other species like the Tiger and Zebra. The striping that occurs in wild boar piglets is most certainly a camouflage pattern (Figure 1C) and it is plausible that selection against this pattern facilitated early animal husbandry.

This study provides an interesting insight into a long-standing evolutionary question. The dramatic phenotypic differences between wild and domestic taxa could either be the result of altered selection upon pre-existing variation in the wild ancestor once under human control, or the result of selection for new mutations with major effects that arise after the domestication process has begun. The *MC1R* locus in pigs provides a clear example of the latter case. Specifically, the *E^P^* allele provides particularly strong evidence for this since it involves two mutations with distinct phenotypic effects: the D124N missense mutation causes dominant black color, and the two base pair insertion at codon 22 causes a frameshift that results in inactivation of the *MC1R* locus. The combination of these two mutations leads to a unique allele and resulting coat color, neither of which could have evolved in a wild context since the appearance of either of the two mutations would have been eliminated from the population by purifying selection. The additive nature of mutations found within domestic *MC1R* alleles proves that shifts in selection pressure can lead to rapid phenotypic change as newly favored alleles form the templates on which additional mutations can be added to create novel phenotypic effects.

It is common knowledge that there has been selection acting on coat color phenotypes in domestic animals since color has been used as breed characteristics. However, breed formation is a very recent phenomenon (within the last few hundred years) and the possibility that coat color variants have been accumulating due to relaxed selection since domestication and then been under strong selection during breed formation has not been excluded. This study demonstrates that selection on coat color phenotypes has a much older history and must trace back to the early period of domestication.

## Materials and Methods

### Sample Location and Collection

DNA samples from 68 unrelated domestic pigs (no common grandparents) were included in the study. The material comprised 45 European pigs representing 31 populations and 23 Chinese pigs representing 19 breeds. The Chinese pigs included members from each of the six traditional types of Chinese indigenous pig breeds described by Zhang [25]. In addition, three Chinese wild boars from two different regions and 12 European wild boars from Poland were included. Genomic DNA was extracted from blood by standard methods or from hair roots by Chelex extraction.

### Sequencing

An *MC1R* fragment including the entire coding region plus 43 bp of 5′-UTR and 208 bp of 3′-UTR was amplified with the forward *EPIG16* primer [7] and the reverse primer PCR2 (5′-CGCCGTCTCTCCAGCCTCCCCCACTC-3′). PCR was carried out on a DNA Engine Gradient Cycler (Perkin Elmer, Norwalk, Connecticut, USA) in a total volume of 35 µl containing 70 ng genomic DNA, 0.5 mM of both primers, 0.3 mM dNTPs, 1× PCR Gold Buffer, 2 U *AmpliTaq Gold* enzyme (Perkin Elmer, Norwalk, Connecticut, USA), 1 M Betaine (SIGMA, Missouri, USA) and 2.5 mM MgCl_2_. The PCR profile was 45 cycles of 45 s each at 94°C, 63.5°C and 72°C; the cycles were preceded by 7 min at 94°C and terminated with 10 min at 72°C. Three other sequencing primers (*Seq1*: 5′-GTCCATCTTCTACGCGCT-3′; *Seq2*: 5′-GGTGGTAGTAGGCGATGA-3′ and *Seq3*: 5′-GGT TCT TGG CGA TGG CGG –3′) were designed to produce a final 963/965 bp sequence including the entire *MC1R* coding region.

The sequences from one sample were compiled into a single contiguous fragment using SEQUENCHER (Gene Codes, Ann Arbor, Michigan, USA). Ambiguous positions were verified by resequencing. The haplotypes carried by heterozygous animals were deduced by sequencing cloned PCR products. Heterozygosity for the *E^p^* mutation comprising a two bp insertion was further verified by fragment analysis as previously described [7].

### Phylogenetic Methods – dN/dS Calculation

A network tree of the MC1R sequences was constructed by hand. The relative frequencies of non-synonymous (dN) and synonymous (dS) substitutions were calculated using the Nei-Gojobori method in MEGA4 [26],[27]. Standard errors were estimated with a bootstrap procedure (500 replicates) and the statistical significance of differences in dN and dS values was calcuated with a Z test [27],[28].

### Nomenclature

We propose a new more informative nomenclature for porcine *MC1R* alleles (Table S1). The nomenclature is composed of four digits where the first two digits are used to distinguish alleles that show documented phenotypic differences and corresponds to the five previously described alleles. The two last digits are used to distinguish alleles presumed to be associated with the same phenotype.

### Accession Numbers

All novel sequences were submitted to GenBank with reference numbers EU443644-EU443726.

## Supporting Information

Table S1. New and old nomenclature for porcine *MC1R/Extension (E)* alleles.
(0.01 MB PDF)

Table S2. Sequence alignment of pig *MC1R/E* alleles. A dash (-) indicates identity to the master sequence.
(0.04 MB PDF)

Table S3. *MC1R* and *Extension* genotypes among all tested pigs.
(0.02 MB PDF)

Table S4. *Extension/MC1R* allele frequencies among European and Asian pigs.
(0.01 MB PDF)
