# Supplementary material for: Contrasting Mode of Evolution at a Coat Color Locus in Wild and Domestic Pigs
Source: PLoS Genet. 2009 Jan 16;5(1):e1000341. doi: 10.1371/journal.pgen.1000341 (PMC2613536; doi:10.1371/journal.pgen.1000341)
Supplement: Table S1. — New and old nomenclature for porcine MC1R/Extension (E) alleles. (0.01 MB PDF) [file pgen.1000341.s001.pdf]

Table S1. New and old nomenclature for porcine *MC1R/ Extension* (*E*) alleles

New	Old[1]	Origin
E^{+} - *Wild-type*		
0101	*1*	European
0102	*5*	Asian
0103	-	Asian
0104	-	Asian
0105	-	Asian
E^{D1}- *Dominant black*		
0201	*2*	Asian
0202	*7*	Asian
0203	-	Asian
E^{D2}- *Dominant black*		
0301	*3*	European
e – Recessive red		
0401	*4*	European
E^{P} – *Black spotting*		
0501	*6*	European
0502	-	European
0503	-	European

[1]References: (1): Kijas JMH, et al. (1998) Genetics 150: 1177-1185; (2): Kijas JMH, et al. (2001) Genetics 158: 779-785; (3): Giuffra E, et al. (2000) Genetics 154: 1785-1791; Gustafsson AC, et al. (2001) Anim Biotech 12: 145-153.
