# Supplementary material for: Contrasting Mode of Evolution at a Coat Color Locus in Wild and Domestic Pigs
Source: PLoS Genet. 2009 Jan 16;5(1):e1000341. doi: 10.1371/journal.pgen.1000341 (PMC2613536; doi:10.1371/journal.pgen.1000341)
Supplement: Table S2. — Sequence alignment of pig MC1R/E alleles. A dash (-) indicates identity to the master sequence. (0.04 MB PDF) [file pgen.1000341.s002.pdf]

Table S2. Sequence alignment of pig *MC1R/E* alleles. A dash (-) indicates identity to the master sequence.

	Codon													
MC1R/E	4	17	21	22	95	102	117	121	122	124	164	166	243	301
0101/ E^{+}	C T T	G C G	G C C	C C C	G T G	C T G	C A G	A A T	G T C	G A C	G C G	C G G	G C G	T A C
	Leu	Ala	Ala	Pro	Val	Leu	Gln	Asn	Val	Asp	Ala	Arg	Ala	Tyr
0102[1]/ E^{+}	. . .	. . .	. . .	. . .	. . .	. . .	. . .	. . C	. . .	. . .	. . .	. . .	. . .	. . .
	—	—	—	—	—	—	—	—	—	—	—	—	—	—
0103/ E^{+}	. . C	. . .	. . .	. . .	. . .	. . .	. . .	. . C	. . .	. . .	. . .	. . .	. . .	. . .
	—	—	—	—	—	—	—	—	—	—	—	—	—	—
0104/ E^{+}	. . .	. . A	. . .	. . .	. . .	. . .	. . .	. . C	. . .	. . T	. . .	. . .	. . A	. . T
	—	—	—	—	—	—	—	—	—	—	—	—	—	—
0105/ E^{+}	. . .	. . A	. . .	. . .	. . .	. . .	. . A	. . C	. . .	. . .	. . .	. . .	. . A	. . T
	—	—	—	—	—	—	—	—	—	—	—	—	—	—
0201/ E^{D1}	. . .	. . A	. . .	. . .	A . .	. C .	. . .	. . C	. . .	. . .	. . .	. . .	. . A	. . .
	—	—	—	—	Met	Pro	—	—	—	—	—	—	—	—
0202/ E^{D1}	. . .	. . A	. . .	. . .	A . .	. C .	. . .	. . C	A . .	. . .	. . .	. . .	. . A	. . .
	—	—	—	—	Met	Pro	—	—	Ile	—	—	—	—	—
0203/ E^{D1}	. . .	. . A	. . .	. . .	A . .	. C .	. . .	. . C	. . .	. . T	. . .	. . .	. . A	. . .
	—	—	—	—	Met	Pro	—	—	—	—	—	—	—	—
0301/ E^{D2}	. . .	. . .	. . .	. . .	. . .	. . .	. . .	. . .	. . .	A . .	. . .	. . .	. . .	. . .
	—	—	—	—	—	—	—	—	—	Asn	—	—	—	—
0401/ *e*	. . .	. . .	. . .	. . .	. . .	. . .	. . .	. . .	. . .	. . .	. T .	. . .	A . .	. . .
	—	—	—	—	—	—	—	—	—	—	Val	—	Thr	—
0501/ E^{P}	. . .	. . .	. . .	+CC	. . .	. . .	. . .	. . .	. . .	A . .	. . .	. . .	. . .	. . .
	—	—	—	FS[2]	—	—	—	—	—	Asn	—	—	—	—
0502/ E^{P}	. . .	. . .	A . .	+CC	. . .	. . .	. . .	. . .	. . .	A . .	. . .	. . .	. . .	. . .
	—	—	Thr	FS[2]	—	—	—	—	—	Asn	—	—	—	—

0503/ *E*^{P}	. . .	. . .	. . .	+CC	. . .	. . .	. . .	. . .	. . .	A . .	. . .	T . .	. . .	. . .
	—	—	—	FS[2]	—	—	—	—	—	Asn	—	Trp	—	—

[1]This allele was previously identified in a Japanese wild boar (Giuffra E, et al. (2000) Genetics 154: 1785-1791) but was not detected in the present study.

[2]Sequence out of frame after codon 22.
