# Supplementary material for: Contrasting Mode of Evolution at a Coat Color Locus in Wild and Domestic Pigs
Source: PLoS Genet. 2009 Jan 16;5(1):e1000341. doi: 10.1371/journal.pgen.1000341 (PMC2613536; doi:10.1371/journal.pgen.1000341)
Supplement: Table S3. — MC1R and Extension genotypes among all tested pigs. (0.02 MB PDF) [file pgen.1000341.s003.pdf]

Table S3. *MC1R* and *Extension* genotypes among all tested pigs

Population (country)[1]	n	Coat color phenotype	*Extension* genotype[2]	*MC1R* genotype
Local European breeds				
Mangalica (Germany)	1	Variable	E^{+}/E^{+}	*0101/0101*
Angler Sattelschwein (Germany)	1	Black with white belt	E^{D2}/E^{D2}	*0301/0301*
Bisaro (Portugal)	1	Red with black spots	E^{D2}/E^{D2}	*0301/0301*
Large Black (UK)	1	Black	E^{D2}/E^{D2}	*0301/0301*
Negro Canario (Spain)	1	Black	E^{D2}/E^{D2}	*0301/0301*
Berkshire (UK)	2	Black with six white points	E^{P}/E^{P}	*0501/0501*
Linderödssvin (Sweden)	5	White/red with black spots	E^{P}/E^{P}	*0501/0501*
Middle White (UK)	1	White	E^{P}/E^{P}	*0501/0501*
Tamworth (UK)	2	Red	E^{P}/E^{P}	*0501/0501*
Leicoma (UK)	1	White	*e/e*	*0401/0401*
British Lop (UK)	1	White	E^{P}/E^{P}	*0502/0502*
Bunte Bentheimer (Germany)	1	White with black spots	E^{P}/E^{P}	*0503/0503*
Créole (France)	1	Black	E^{D1}/E^{D2}	*0201/0301*
	1	Black	E^{D2}/E^{P}	*0301/0501*
	1	Black	E^{D2}/e	*0301/0401*
**Commercial European populations**				
Hampshire 02 (Germany)	1	Black with white belt	E^{D2}/E^{D2}	*0301/0301*
Landrace 05 (Denmark)	1	White	E^{P}/E^{P}	*0501/0501*
Landrace 09 (Iceland)	1	White	E^{P}/E^{P}	*0501/0501*
Landrace 10 (UK)	1	White	E^{P}/E^{P}	*0501/0501*
Landrace 11 (UK)	2	White	E^{P}/E^{P}	*0501/0501*
Landrace 12 (UK)	3	White	E^{P}/E^{P}	*0501/0501*

Landrace 14 (Germany)	1	White	E^P/E^P	*0501/0501*
Large White 05 (UK)	1	White	E^P/E^P	*0501/0501*
Large White 06 (UK)	2	White	E^P/E^P	*0501/0501*
Large White 07(UK)	1	White	E^P/E^P	*0501/0501*
Pietrain 03 (Germany)	1	White with black spots	E^P/E^P	*0501/0501*
Pietrain 04 (UK)	1	White with black spots	E^P/E^P	*0501/0501*
Duroc 02 (Germany)	1	Red	e/e	*0401/0401*
Duroc 03 (UK)	1	Red	e/e	*0401/0401*
Landrace 04 (Denmark)	1	White	E^P/E^P	*0502/0502*
Landrace 08 (Norway)	1	White	E^P/E^P	*0502/0503*
Large White 02 (Germany)	3	White	E^P/E^P	*0501/0501*
	1	White	E^P/E^P	*0503/0503*
European wild boar				
Wild boar (Poland)	12	Wild-type	E^+/E^+	*0101/0101*
Chinese pig breeds				
Bamei	1	Black	E^{D1}/E^{D1}	*0201/0201*
Huzhu	2	Black	E^{D1}/E^{D1}	*0201/0201*
Jiangquhai	1	Black	E^{D1}/E^{D1}	*0201/0201*
Meishan (UK)	1	Black	E^{D1}/E^{D1}	*0201/0201*
Laiwu	1	Black	E^{D1}/E^{D1}	*0201/0201*
Leping	1	Black	E^{D1}/E^{D1}	*0201/0201*
Rongchang	2	White with black spots around eyes	E^{D1}/E^{D1}	*0201/0201*
Neijiang	2	Black	E^{D1}/E^{D1}	*0201/0201*
Qianbei	1	Black	E^{D1}/E^{D1}	*0201/0201*
Shanggao	1	White with two-end-black	E^{D1}/E^{D1}	*0201/0201*
Tongcheng	1	White with two-end-black	E^{D1}/E^{D1}	*0201/0201*

Xiang	1	Black	E^{D1}/E^{D1}	*0201/0201*
Yimeng	1	Black	E^{D1}/E^{D1}	*0201/0201*
Yushan	1	Black	E^{D1}/E^{D1}	*0201/0201*
Zang	1	Black	E^{D1}/E^{D1}	*0201/0201*
Jinhua	1	White with two-end-black	E^{D1}/E^{D1}	*0203/0203*
Xiangxi	1	Black	E^{D1}/e	*0201/0401*
Shengxian Spotted	1	Black with white spots	E^{D1}/E^{D1}	*0201/0202*
Min	1	Black	E^{D1}/E^{P}	*0202/0501*
Jiaozhou	1	Black	E^{D1}/E^{P}	*0201/0502*
Chinese wild boar				
Hainan wild boar	1	Wild	E^{+}/E^{+}	*0103/0103*
	1	Wild	E^{+}/E^{+}	*0104/0105*
Dongbei wild boar	1	Wild	E^{+}/E^{P}	*0105/0501*

[1]Country of origin represents where the sampled pigs were located but the breed may originate from another country. For instance, Meishan (UK) represents a population of the Chinese Meishan breed kept in the UK. All samples from Europe are from the PigBioDiv panel (see http://www.projects.roslin.ac.uk/pigbiodiv/ for further details)

[2]Deduced based on coat color and *MC1R* sequence
