# Supplementary material for: Contrasting Mode of Evolution at a Coat Color Locus in Wild and Domestic Pigs
Source: PLoS Genet. 2009 Jan 16;5(1):e1000341. doi: 10.1371/journal.pgen.1000341 (PMC2613536; doi:10.1371/journal.pgen.1000341)
Supplement: Table S4. — Extension/MC1R allele frequencies among European and Asian pigs. (0.01 MB PDF) [file pgen.1000341.s004.pdf]

Table S4. *Extension*/*MC1R* allele frequencies among European and Asian pigs

Allele	European		Asian	
	Domestic<br>n=45	Wild<br>n=12	Domestic<br>n=23	Wild<br>n=3
E^{+}	.02	1.00	0	.83
E^{D1}	.01	0	.94	0
E^{D2}	.14	0	0	0
e	.08	0	.02	0
E^{P}	.75	0	.04	.17
Total	1.00	1.00	1.00	1.00
